# Clinical manifestations, prognostic factors, and outcomes of adenovirus pneumonia after allogeneic hematopoietic stem cell transplantation

**DOI:** 10.1186/s12985-024-02383-1

**Published:** 2024-05-14

**Authors:** Yuewen Wang, Xiaohui Zhang, Lanping Xu, Yu Wang, Chenhua Yan, Huan Chen, Yuhong Chen, Fangfang Wei, Wei Han, Fengrong Wang, Jingzhi Wang, Xiaojun Huang, Xiaodong Mo

**Affiliations:** 1 Beijing Key Laboratory of Hematopoietic Stem Cell Transplantation, Peking University People’s Hospital, Peking University Institute of Hematology, National Clinical Research Center for Hematologic Disease, No. 11 Xizhimen South Street, Xicheng District, Beijing, 100044 China; 2 Research Unit of Key Technique for Diagnosis and Treatments of Hematologic Malignancies, Chinese Academy of Medical Sciences, Beijing, 2019RU029 China; 3 Peking-Tsinghua Center for Life Sciences, Beijing, 100871 China

**Keywords:** Adenovirus pneumonia, Posttransplantation, Allogeneic hematopoietic stem cell transplantation

## Abstract

**Background:**

Severe pneumonia is one of the most important causes of mortality after allogeneic hematopoietic stem cell transplantation (allo-HSCT). Adenovirus (ADV) is a significant cause of severe viral pneumonia after allo-HSCT, and we aimed to identify the clinical manifestations, prognostic factors, and outcomes of ADV pneumonia after allo-HSCT.

**Methods:**

Twenty-nine patients who underwent allo-HSCT at the Peking University Institute of Hematology and who experienced ADV pneumonia after allo-HSCT were enrolled in this study. The Kaplan–Meier method was used to estimate the probability of overall survival (OS). Potential prognostic factors for 100-day OS after ADV pneumonia were evaluated through univariate and multivariate Cox regression analyses.

**Results:**

The incidence rate of ADV pneumonia after allo-HSCT was approximately 0.71%. The median time from allo-HSCT to the occurrence of ADV pneumonia was 99 days (range 17–609 days). The most common clinical manifestations were fever (86.2%), cough (34.5%) and dyspnea (31.0%). The 100-day probabilities of ADV-related mortality and OS were 40.4% (*95% CI* 21.1%-59.7%) and 40.5% (95% *CI* 25.2%-64.9%), respectively. Patients with low-level ADV DNAemia had lower ADV-related mortality and better OS than did those with high-level (≥ 10^6^ copies/ml in plasma) ADV DNAemia. According to the multivariate analysis, high-level ADV DNAemia was the only risk factor for intensive care unit admission, invasive mechanical ventilation, ADV-related mortality, and OS after ADV pneumonia.

**Conclusions:**

We first reported the prognostic factors and confirmed the poor outcomes of patients with ADV pneumonia after allo-HSCT. Patients with high-level ADV DNAemia should receive immediate and intensive therapy.

**Supplementary Information:**

The online version contains supplementary material available at 10.1186/s12985-024-02383-1.

## Introduction

Allogeneic hematopoietic stem cell transplantation (allo-HSCT) is the most significant curative method for treating hematologic malignancies [1, 2]; however, posttransplant infection can cause severe morbidity and mortality after allo-HSCT. Severe pneumonia is a critical infectious complication after allo-HSCT, and the mortality rate can reach nearly 70% [3].

The cytomegalovirus is the most common pathogen causing severe pneumonia in allo-HSCT recipients [4]. For example, Aguilar-Guisado et al. [5] reported that cytomegalovirus (CMV) was the most common pathogen causing viral pneumonia after allo-HSCT, and Cao et al. [6] demonstrated that viral infections, especially CMV, Epstein–Barr virus (EBV), and respiratory syncytial virus (RSV), accounted for almost half of all cases of pathogen-positive severe pneumonia. In addition, although some viruses are not common pathogens in severe pneumonia [3, 5], they can lead to life-threatening outcomes, and adenovirus (ADV) is a good example of such infection.

ADV is an important viral pathogen of upper respiratory tract infection in patients with normal immune function [7–9] and is a benign and self-curing disease. However, in immunocompromised individuals, ADV infection can affect many organs and can result in fatal outcomes [10, 11]. ADV pneumonia is the most severe manifestation of ADV infection. Ohori et al. [12] reported that all 4 patients diagnosed with ADV pneumonia died within 50 days after lung transplantation. Similarly, in children who underwent liver transplantation, the mortality rate was as high as 75% [13]. However, most studies have reported only a small sample (*n* < 5) of allo-HSCT recipients with ADV pneumonia [3, 5, 14]. Although Yilmaz et al. [15] reported that 18 patients in a cohort with adenoviral infection had pneumonia, they did not further describe the characteristics of these patients in these subgroups. Thus far, no study has focused on a disease-specific cohort of patients with ADV pneumonia after allo-HSCT, and the clinical manifestations, prognostic factors, and outcomes of these patients are still unknown.

Therefore, in the present study, we aimed to identify the clinical manifestations, prognostic factors, and outcomes of ADV pneumonia after allo-HSCT.

## Patients and methods

### Patients

This study was conducted based on the transplant database of Peking University, Institute of Hematology (PUIH), and the inclusion criteria were as follows: (1) received allo-HSCT between May 1st, 2019, and October 1st, 2023; and (2) were diagnosed with ADV pneumonia after allo-HSCT. The last follow-up for survivors was December ^4^, 2023. The study was conducted in accordance with the *Declaration of Helsinki*, and the protocol was approved by the Institutional Review Board of Peking University People’s Hospital.

### Transplant regimen

The major preconditioning regimens included cytarabine, busulfan, cyclophosphamide, semustine, and antithymocyte globulin (ATG) [16, 17]. The protocols for graft-versus-host disease (GVHD) and infection prophylaxis were previously reported [18–21].

### Detection of ADV

ADV was detected via real-time quantitative polymerase chain reaction (RT–PCR) in plasma and other samples, e.g., bronchoalveolar lavage fluid (BALF) [22]. While most of the patients had quantitative ADV results, some patients had only semiquantitative results. Semiquantitative PCR refers to a PCR technique developed from traditional qualitative analysis methods and capable of measuring the relative quantity of target products [23]. Based on the results of semiquantitative PCR, it was possible to determine the level of ADV positivity according to the cycle threshold (CT) values of the samples. A CT value above 34 indicated that the virus load was less than 10^3^ copies, while a CT value less than 26 indicated that the virus load was greater than 10^6^ copies. All the samples were transported to the laboratory within 3 h. RT‒PCR was performed on an ABI-7500 (Applied Biosystems, USA).

### Diagnosis of ADV pneumonia

We classified ADV pneumonia as proven or probable. Proven ADV pneumonia was diagnosed by pathological sputum production, hypoxia, or pulmonary infiltrates together with identification of ADVs in respiratory secretions, preferentially in samples taken from the sites of involvement. Probable ADV pneumonia was diagnosed as new onset respiratory presentation and pulmonary infiltrates, together with identification of ADVs in samples in addition to respiratory secretions (e.g., plasma) but in the absence of other documented causes of pneumonia.

### Definition

The primary endpoint was ADV-related mortality, which was defined as the mortality associated with ADV infection either directly or indirectly. The secondary endpoint was overall survival (OS), which was defined as the time from pneumonia to death from any cause or the date of last contact. GVHD was diagnosed and graded according to international recommendations [24]. The immunodeficiency score index (ISI) was calculated as previously described [25].

### Statistical analysis

The data were censored at the time of death or the last available follow-up. Continuous variables were compared with the Mann–Whitney *U* test; categorical variables were compared with the *χ*^2^ test and Fisher’s exact test. The Kaplan–Meier method was used to estimate the probability of OS. Potential prognostic factors for outcomes after ADV pneumonia were evaluated through univariate and multivariate Cox regression analyses. All factors with *P* < 0.1 in the univariate analysis were included in the multivariate regression, and *P* < 0.05 was considered to be statistically significant. All reported *P* values were based on two-sided tests. Data analyses were conducted with SPSS software (SPSS, Inc., Chicago, IL, USA). R statistical software was used for competing risk analysis.

## Results

### Patient characteristics

Twenty-nine cases with ADV pneumonia were identified from a cohort of 4083 patients who underwent allo-HSCT between May 1st, 2019 to October 1st, 2023 (0.71%), and their characteristics were shown in Table 1. All of them achieved neutrophil engraftment, and 72.4% (*n* = 21) of them achieved platelet engraftment. Eleven (37.9%) and seven (24.1%) patients experienced acute GVHD and chronic GVHD, respectively, before ADV pneumonia. The median follow-up was 52 days (range 9-697 days) after the occurrence of ADV pneumonia.

**Table 1:** Patient characteristics

Characteristics	All	Survival	Death	*P* value
Patients, n	29	10	19	
Age, median (range)	35 (11–63)	43.5 (25–61)	34 (11–63)	0.581
Sex, n (%)				0.494
Male	17 (58.6%)	5 (50%)	12 (63.2%)	
Female	12 (41.4%)	5 (50%)	7 (36.8%)	
Diagnosis (%)				0.093
ALL	10 (34.5%)	1 (10.0%)	9 (47.4%)	
AML	10 (34.5%)	6 (60.0%)	4 (21.1%)	
MDS	4 (13.8%)	2 (20.0%)	2 (10.4%)	
Others	5 (17.2%)	1 (10.0%)	4 (21.1%)	
Disease status, n (%)				0.044
CR1	19 (65.5%)	9 (90.0%)	10 (52.6%)	
≥CR2	10 (34.5%)	1 (10.0%)	9 (47.4%)	
Transplant graft, n (%)				0.127
BM + PBSC	5 (17.2%)	0	5 (26.3%)	
PBSC	21 (72.4%)	8 (80.0%)	13 (68.4%)	
Other	3 (10.4%)	2 (20.0%)	1 (5.3%)	
Donor–recipient gender match, n (%)				0.619
Male–male	9 (31.0%)	4 (40.0%)	5 (26.3%)	
Male–female	8 (27.6%)	3 (30.0%)	5 (26.3%)	
Female–male	7 (24.1%)	1 (10.0%)	6 (31.6%)	
Female–female	4 (13.8%)	2 (20.0%)	2 (10.5%)	
Other (only cord blood infused)	1 (3.4%)	0	1 (5.3%)	
Number of HLA-A, B, DR mismatches, n (%)				0.568
1	1 (3.4%)	0	1 (5.3%)	
2	1 (3.4%)	0	1 (5.3%)	
3	27 (93.1%)	10 (100%)	17 (89.5%)	
Donor-recipient relationship, n (%)				0.747
Father-child	10 (34.5%)	3 (30.0%)	7 (36.8%)	
Mother-child	5 (17.2%)	1 (10.0%)	4 (21.1%)	
Sibling-sibling	6 (20.7%)	3 (30.0%)	3 (15.8%)	
Child-parent	7 (24.1%)	3 (30.0%)	4 (21.1%)	
Other	1 (3.4%)	0	1 (5.3%)	
Infused cell dose, median (range)				
MNC (× 10^8^/kg)	9.76 (5.56–17.72)	12.43 (7.46–17.72)	8.70 (5.56–15.61)	0.009
CD34^+^ cells (× 10^6^/kg)	3.23 (1.21–7.43)	3.10 (1.21–7.09)	4.16 (1.38–7.43)	0.890
Engraftment (yes or no), n (%)				
Neutrophils	29 (100%)	10 (100%)	19 (100%)	NS
Platelets	21 (72.4%)	8 (80.0%)	13 (68.4%)	0.507
Acute GVHD prior to ADV pneumonia, n (%)				0.564
None	18 (62.1%)	6 (60.0%)	12 (63.2%)	
Grade I	6 (20.7%)	3 (30.0%)	3 (15.7%)	
Grade II-IV	5 (17.2%)	1 (10.0%)	4 (21.1%)	
Chronic GVHD prior to pneumonia, n (%)				0.181
Total	7 (24.1%)	4 (40.0%)	3 (15.8%)	
Clinical extensive	4 (13.8%)	3 (30.0%)	1 (5.3%)	

**Abbreviations**: ALL = acute lymphoblastic leukemia; AML = acute myeloid leukemia; MDS = myelodysplastic syndromes; CR = complete remission; BM = bone marrow; PBSC = peripheral blood stem cell; MNC = mononuclear cells; HLA = human leukocyte antigen; GVHD = graft-versus-host disease.

### Characteristics of patients with ADV pneumonia

The median time from allo-HSCT to the occurrence of ADV pneumonia was 99 days (range 17–609 days). Five (17.2%) and twenty-four (82.8%) patients were diagnosed with proven and probable ADV pneumonia, respectively. The clinical manifestations are summarized in Table 2. Twenty-five patients (86.2%) developed fever when ADV pneumonia occurred, with a median temperature of 38.2 °C (range 37.2–39.4 °C). Twenty (69.0%) patients underwent arterial blood gas analysis; 17 (85%) had hypoxemia, and 11 (64.7%) had type I respiratory failure.

**Table 2:** Characteristics of ADV pneumonia after transplantation

Characteristics	All	Probable ADV pneumonia	Proven ADV pneumonia	*P* value
Patients, n	29	24	5	
Median time of ADV pneumonia post-transplantation, d, (range)	99 (17–609)	93 (17–609)	217 (52–296)	0.149
Median follow-up days after ADV pneumonia, (range)	52 (9-697)	57 (9-697)	24 (13–532)	0.453
Symptoms at diagnosis, n (%)				
Fever	25 (86.2%)	21 (87.5%)	4 (80.0%)	0.658
Cough	10 (34.5%)	6 (25.0%)	4 (80.0%)	0.019
Expectoration	4 (13.8%)	1 (4.2%)	3 (60.0%)	0.001
Dyspnea	9 (31.0%)	5 (20.8%)	4 (80.0%)	0.009
Chest tightness	3 (10.3%)	3 (12.5%)	0	0.404
Median maximum temperature after diagnosis, °C (range)	38.0 (36.0-39.4)	38.0 (36.0-39.4)	38.2 (36.0-39.2)	0.506
Median maximum breaths per minute after diagnosis, n (range)	20 (18–42)	20 (18–42)	20 (19–40)	0.378
Number of immunosuppression agents used before ADV pneumonia, n (%)				0.347
1	9 (31.0%)	6 (25.0%)	3 (60.0%)	
2	12 (41.4%)	10 (41.7%)	2 (40.0%)	
3	7 (24.1%)	7 (29.2%)	0	
4	1 (3.4%)	1 (4.2%)	0	
Chest CT, n (%)				
Patch	22 (75.9%)	17 (70.8%)	5 (100%)	0.166
Nodule	15 (51.7%)	13 (54.2%)	2 (40.0%)	0.564
Ground-glass opacity	15 (51.7%)	14 (58.3%)	1 (20.0%)	0.119
Fibrous stripes	10 (34.5%)	9 (37.5%)	1 (20.0%)	0.454
Pleural effusion	15 (51.7%)	13 (54.2%)	2 (40.0%)	0.564
Consolidation opacity	7 (24.1%)	4 (16.7%)	3 (60.0%)	0.039
Laboratory values at ADV pneumonia onset, median (range)				
Absolute monocyte count, 10^9^/L	0.22 (1.00–2.00)	0.17 (0–2.00)	0.5 (0.10–1.40)	0.191
Albumin, g/L	31.3 (26.1–37.5)	31.8 (26.8–37.5)	30.8 (26.1–34.2)	0.644
LDH, U/L	372 (112–1427)	372.5 (112–1427)	298 (149–507)	0.908
BUN, mmol/L	5.74 (1.00-24.65)	6.49 (1.00-24.65)	4.15 (1.90–5.10)	0.035
C-reactive protein, mg/L	38.5 (0-344.6)	35.3 (0-344.6)	40.1 (15.3–92.5)	0.686
Corticosteroid treatment during ADV pneumonia, n (%)	19 (65.5%)	16 (66.7%)	3 (60.0%)	0.775
Corticosteroid dose, mg/kg, median (range)	1.33 (0.22–9.62)	1.37 (0.38–9.62)	1.00 (0.22–5.36)	0.426
Corticosteroid dose < 2 mg/kg, n (%)	13 (44.8%)	11 (45.8%)	2 (40.0%)	0.943
Corticosteroid used within 7 days after ADV pneumonia onset, n (%)	10 (34.5%)	8 (33.3%)	2 (40.0%)	0.596
Treatment of ADV pneumonia, n (%)				
Intravenous gamma globulin	19 (65.5%)	17 (70.8%)	2 (40.0%)	0.187
Acyclovir	13 (44.8%)	11 (45.8%)	2 (40.0%)	0.811
Cidofovir	11 (37.9%)	9 (37.5%)	2 (40.0%)	0.917
Ganciclovir	5 (17.2%)	4 (16.7%)	1 (20.0%)	0.858
ISI score, n (%)				0.042
0–2 score	3 (10.3%)	1 (4.2%)	2 (40.0%)	
3–6 score	22 (75.9%)	20 (83.3%)	2 (40.0%)	
7–12 score	4 (13.8%)	3 (12.5%)	1 (20.0%)	
ICU administration, n (%)	15 (51.7%)	13 (54.2%)	2 (40.0%)	0.564
Median duration of ICU administration, days (range)	4 (1–38)	3 (1–38)	14 (10–18)	0.304
Need for invasive mechanical ventilation, n (%)	13 (44.8%)	11 (45.8%)	2 (40.0%)	0.811
ADV DNAemia (yes or no), n (%)	25 (86.2%)	23 (95.8%)	2 (40.0%)	0.001
ADV titer in plasma, n (%)				0.001
Negative	4 (13.8%)	1 (4.2%)	3 (60.0%)	
< 10^6^copies/ml	20 (69.0%)	19 (79.2%)	1 (20.0%)	
≥ 10^6^copies/ml	5 (17.2%)	4 (16.6%)	1 (20.0%)	
ADV detected in BALF, n (%)	5 (17.2%)	0	5 (100%)	NS

**Abbreviations**: ADV = adenovirus; CT = computed tomography; BUN = blood urea nitrogen; LDH = lactate dehydrogenase; ISI = immunodeficiency scoring index; ICU = intensive care unit; BALF = bronchoalveolar lavage fluid.

All of the patients underwent chest computed tomography (CT), and only the initial scan was included in this analysis. A patchy appearance was the most common presentation (*n* = 22), followed by ground-glass opacity (*n* = 15), pleural effusion (*n* = 15), nodules (*n* = 15), fibrous stripes (*n* = 10), and consolidation opacity (*n* = 7).

The quantitative or semiquantitative results of 25 (86.2%) patients were indicative of ADV DNAemia. Nineteen patients with ADV DNAemia had median viral titers of 4.06 × 10^4^ copies (range 1.0 × 10^3^ to 1.04 × 10^7^ copies). Six patients had semiquantitative results for ADV DNAemia; 3 patients had < 10^3^ copies, and 3 patients had ≥ 10^6^ copies. We defined a PCR value ≥ 10^6^ copies/ml in plasma as high-level ADV DNAemia, and 5 (20%) patients had high-level ADV DNAemia. The median duration of ADV infection for patients who died and survived was 16 days (range 5–71) days, and 12 days (range 3–30) days, respectively (*P* = 0.377).

Seven patients underwent BAL, and ADV was detected in BALF samples from 5 patients. The quantitative results revealed that the median viral titer was 4.9 × 10^4^ copies (range 1 × 10^3^ to 4.6 × 10^5^ copies). One patient had a semiquantitative result of 10^3^ copies/ml. We defined a PCR value ≥ 10^5^ copies/ml in BALF as high-level ADV PCR positivity in BALF (*n* = 2). In addition, plasma and BALF samples from two patients were simultaneously ADV positive according to PCR.

### Treatment and outcomes after ADV pneumonia

The most common therapies for ADV pneumonia included intravenous gamma globulin (65.5%), acyclovir (44.8%), cidofovir (37.9%), and ganciclovir (17.2%).

Nineteen (65.5%) patients received corticosteroid therapy. Among these patients, 12 (63.2%) of them were administered steroids specifically for ADV pneumonia, and the steroid use for the rest of these patients was due to both exacerbation of GVHD and ADV pneumonia. The initial methylprednisolone (MP) dose was 1.33 mg/kg/d (range 0.22–9.62 mg/kg/d), and the median time from the diagnosis of ADV pneumonia to the beginning of corticosteroid therapy was 4 days (range 0–54 days).

Fifteen (51.7%) patients were admitted to the intensive care unit (ICU). The median time from diagnosis of ADV pneumonia to ICU admission was 10 days (range 0–84 days). Sixteen (55.2%) patients received mechanical ventilation after ICU admission, and 11 and 13 patients received noninvasive mechanical ventilation and invasive mechanical ventilation, respectively. The median duration of ICU admission was 4 days (range 1 to 38 days).

The univariate analysis for ICU admission and invasive mechanical ventilation is shown in Additional file 1. According to the multivariate analysis, high-level ADV DNAemia (HR 5.45, *95% CI* 1.55–19.16; *P* = 0.008) was independently associated with ICU admission. Moreover, it was the only risk factor for invasive mechanical ventilation according to multivariate analysis (HR 5.45, *95% CI* 1.55–19.16; *P* = 0.008).

### Mortality and survival in patients with ADV pneumonia

Nineteen (65.5%) patients died after contracting ADV pneumonia. Eleven patients died from ADV pneumonia, and the median duration from the diagnosis of ADV pneumonia to death was 17 days (range 9–86 days). In addition, 3, 1, 2, and 2 patients died from relapse, GVHD, septic shock, and acute heart failure, respectively.

**Fig. 1:**
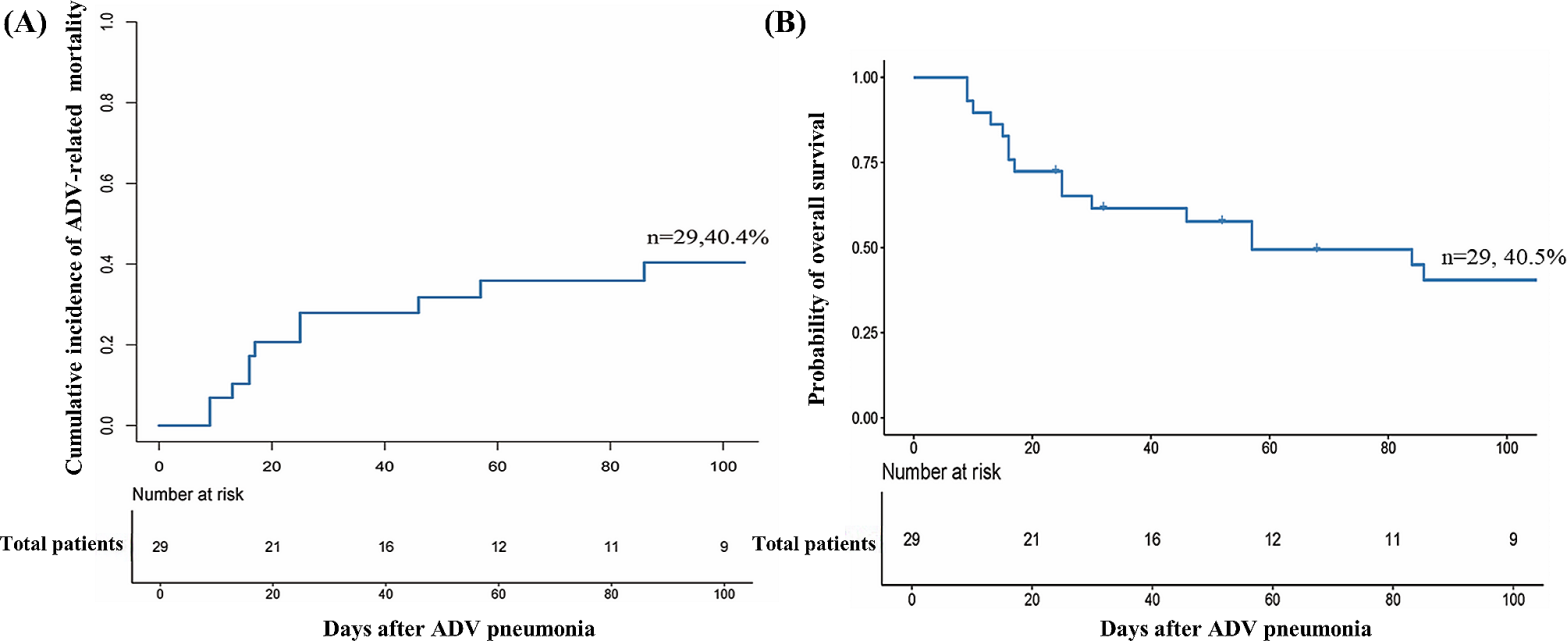
**(A)** ADV-related mortality at 100 days after ADV pneumonia in total patient population. **(B)** Overall survival at 100 days after ADV pneumonia in total patient population

**Fig. 2:**
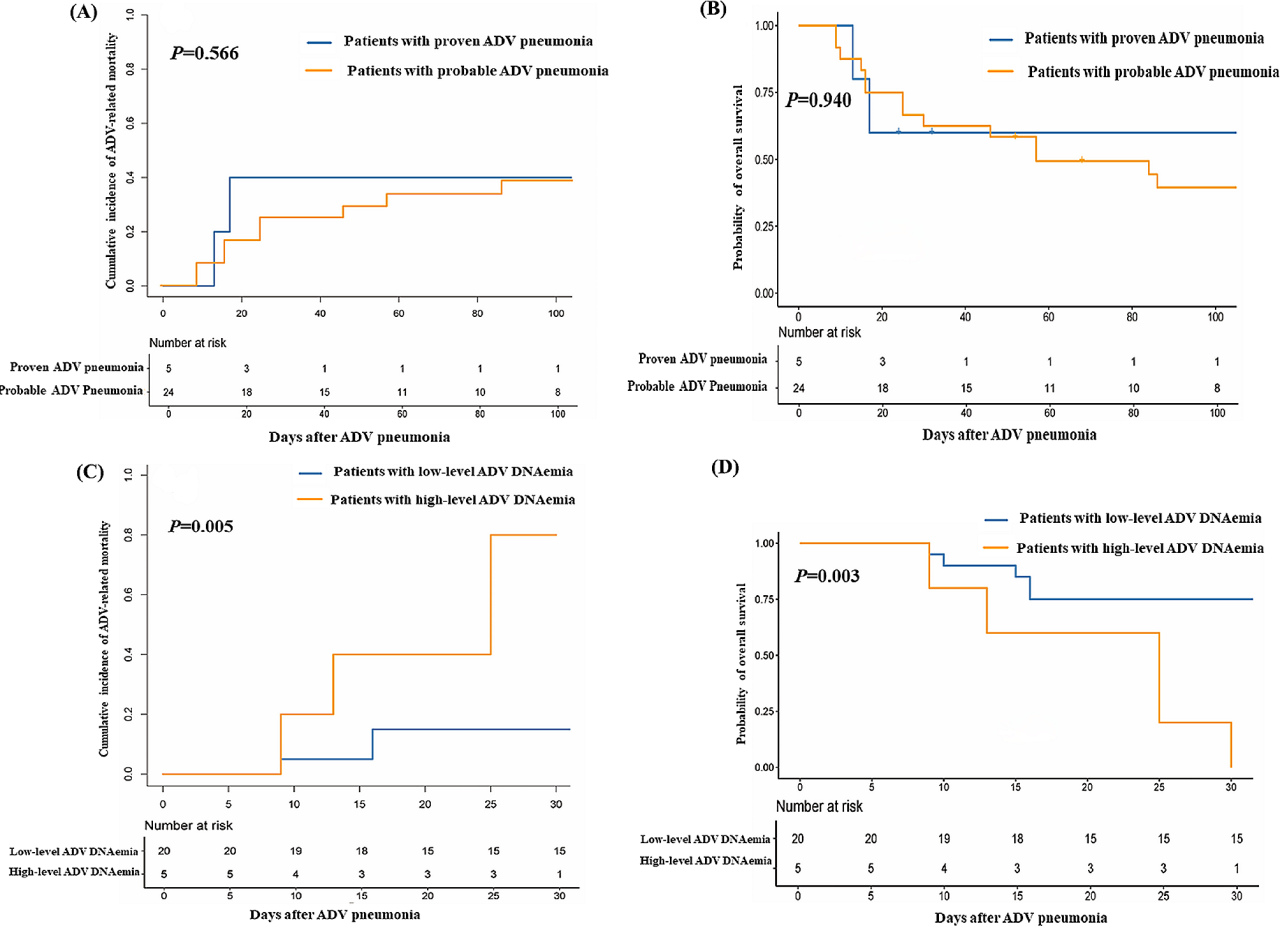
ADV-related mortality at 100 days after ADV pneumonia in **(A)** proven and probable ADV pneumonia and **(C)** high- and low-level ADV DNAemia. Overall survival at 100 days after ADV pneumonia in **(B)** proven and probable ADV pneumonia and **(D)** high- and low-level ADV DNAemia

The 100-day cumulative incidence of ADV-related mortality was 40.4% (*95% CI* 21.1–59.7%) in the total patient population (Fig. 1A) and 40.0% (*95% CI* 0–89.0%) and 38.6% (95% *CI* 18.0–59.2%) (*P* = 0.566) for patients with proven and probable ADV pneumonia, respectively (Fig. 2A). The 30-day cumulative incidence of ADV-related mortality was 15.0% (95% *CI* 0–31.1%) versus 80.0% (95% *CI* 37.5–100%) (*P* = 0.005) for patients with low-level versus high-level ADV DNAemia (Fig. 2C).

The probability of OS at 100 days after the occurrence of ADV pneumonia was 40.5% (95% *CI* 25.2–64.9%) in the total patient population (Fig. 1B) and was 60.0% (95% *CI* 29.3–100%) versus 39.5% (95% *CI* 23.6–66.0%) (*P* = 0.940) for patients with proven versus probable ADV pneumonia (Fig. 2B). The probability of OS at 30 days after the occurrence of ADV pneumonia was 75.0% (95% *CI* 58.2–96.6%) versus 0% (*P* = 0.003) for patients with low-level versus high-level ADV DNAemia (Fig. 2D).

The univariate analysis for ADV-related mortality and OS is shown in Additional file 1. According to the multivariate analysis, a high level of ADV DNAemia was the only risk factor for ADV-related mortality (HR 6.39, *95% CI* 1.43–28.67; *P* = 0.015) and OS at 100 days after the occurrence of ADV pneumonia (HR 5.45, *95% CI* 1.55–19.16; *P* = 0.008).

## Discussion

In the present study, we demonstrated that the incidence rate of ADV pneumonia after allo-HSCT was approximately 0.71%, which was similar to the result of 0.63% reported by Yilmaz et al. [15]. Besides, we observed that the incidence of ADV-related mortality and the probability of OS at 100 days were 40.4% and 40.5%, respectively, for patients with ADV pneumonia after allo-HSCT. Thus far, this was the first and largest study identifying the clinical characteristics of ADV pneumonia in allo-HSCT recipients.

Intensive immunosuppression is a poor prognostic factor for viral disease because it delays viral clearance. Shah et al. [25] established an index (i.e., the ISI) to demonstrate the association between immunosuppression and the outcome of viral pneumonia, which could predict the mortality of patients with RSV pneumonia after allo-HSCT. Although previous studies have indicated that the ISI could predict mortality due to viral pneumonia after allo-HSCT [26–28], some research has reported that the ISI could predict only the risk of progression from upper respiratory tract infection to lower respiratory tract infection [29, 30] and was not associated with survival in patients with viral pneumonia after allo-HSCT [29, 31–33]. In the present study, we observed that the ISI could not predict ADV-related mortality or OS after the occurrence of ADV pneumonia. We noticed that a majority of the ADV pneumonia patients were categorized into the intermediate-risk ISI group; therefore, identifying the association between the ISI and mortality in patients with ADV pneumonia was difficult. This finding suggested that there might be critical differences between RSV and ADV pneumonia in allo-HSCT recipients.

In addition, Cao et al. [6] established a prognostic index for late-onset severe pneumonia after allo-HSCT that included the monocyte count, level of albumin, level of lactic dehydrogenase, and level of blood urea nitrogen. This prognostic index was associated with ICU admission, invasive mechanical ventilation, ADV pneumonia-related mortality, and OS in our univariate analysis, although these differences were not significant according to the multivariate analysis. This finding suggested its potential predictive value in treating ADV pneumonia after allo-HSCT, which is worthy of identification in the future.

We observed that high-level ADV DNAemia, that is, an ADV PCR value ≥ 10^6^ copies/ml in plasma, was the most essential prognostic factor for ADV pneumonia. Other studies have also reported an association between ADV DNAemia and clinical outcomes after allo-HSCT [34–37]. Claas et al. [38] demonstrated that in transplant recipients, a significant increase in ADV loads (e.g., exceeding 10^11^ copies/ml of serum or plasma) could be observed in fatal cases. They also reported that patients with viral loads greater than 10^6^ copies/ml might be considered at risk for fatal complications. Mynarek et al. [37] showed that a peak ADV concentration > 10^4^ copies/mL in the blood was an independent risk factor for poor OS in a cohort of 238 pediatric patients who underwent allo-HSCT. Deambrosis et al. [35] reviewed 62 children with ADV DNAemia after allo-HSCT and reported that a peak viral load > 8 000 copies/ml in blood was significantly associated with increased nonrelapse mortality, and relapse-free survival was only 40%. As supported by the findings of previous publications, clearing the virus was the most crucial therapy for controlling this disease after allo-HSCT [39–41]. Thus, immediate and intensive therapies should be applied for pneumonia patients with high-level ADV DNAemia.

In terms of antiviral therapies, acyclovir is one of the conventional drugs for preventing viral infections in transplant patients in our center. Ganciclovir was previously reported to be effective against ADV in vitro [42]. Bruno et al. [43] showed that the administration of ganciclovir was related to a significantly lower risk of developing ADV infections in transplant recipients. Cidofovir is one of the most important treatments for ADV infection and can suppress all subtypes of ADV in vitro [44]. However, in China, cidofovir is classified as special management drug, and the use of this drug required strict regulations and procedures to be followed. The sales and use of cidofovir could only be obtained through special approval procedures when needed clinically, and could only be used by approved hospitals or professional research institutions. Therefore, the application of cidofovir was relatively limited. In our center, if patients could purchase cidofovir, we would recommend them to use this drug as soon as possible. If not, the use of gamma globulin as well as acyclovir/ganciclovir would be recommended. It was reported that cidofovir could help to clear ADV in nonimmunocompromised patients with ADV pneumonia [45]. However, recent research has indicated that the efficacy of cidofovir is not satisfactory in patients with disseminated ADV infections after allo-HSCT [46, 47]. For instance, Inamoto et al. [48] reported that although cidofovir was used in 119 patients with viremia or disseminated disease after allo-HSCT, the probabilities of physician-assessed improvement and OS were only 59% and 28%, respectively. Similarly, in the present study, the incidence of ADV-related mortality in patients with ADV pneumonia was as high as 40%. Therefore, more effective therapies should be identified for these patients.

Virus-specific cytotoxic T cells, such as CMV and EBV cytotoxic T cells, have been infused into patients with different viral infections after allo-HSCT [49–51]. Feuchtinger et al. [52] performed ADV-specific T-cell immunotherapy in 9 pediatric patients with systemic ADV infection after allo-HSCT, and 1.2–50 × 10^3^/kg T cells were infused into these patients. Feuchtinger et al. reported a significant decrease in viral DNA in the peripheral blood and stool in 5 of 6 evaluable patients with an in vivo expansion of specific T cells, suggesting that this new treatment might be effective. In addition, Taniguchi et al. [47] treated 4 patients with a combination of cidofovir and unmanipulated low-dose donor lymphocyte infusion, and all of these patients survived, indicating that cytotherapy might be a potentially efficient treatment for ADV pneumonia. This topic is worth exploring in future research.

In our previous studies, we observed that patients who received late (≤ 1 week after pneumonia diagnosis) or low-dose corticosteroid therapy (MP ≤ 2 mg/kg/day) had the best outcomes, and their OS rate reached 60% [53]. We subsequently prevented the use of early and high-dose corticosteroid therapy in patients with severe pneumonia after allo-HSCT. In the present study, only 10 and 6 patients, respectively, received MP therapy within 1 week after pneumonia diagnosis and received high-dose MP. However, these factors did not help to further improve the outcomes of ADV pneumonia patients. Thus, the optimal dose and timing of corticosteroid treatment should be further studied in these patients.

In the present study, the median age of patients who survive and did not survive was 45 and 34 years old, respectively. Age might be one of the commonly recognized risk factors for ADV disease in allo-HSCT recipients [54, 55], and Bruno et al. [43] reported that younger age (*P* = 0.001) was one of the risk factors for ADV infection after allogeneic and autologous transplantation. However, patients’ age was not associated with clinical outcomes in the present study. Considering the high mortality and small sample of survivors, we could not further identify the association between age and the clinical outcomes, which should be further studied by large sample, prospective studies.

This study has several limitations. Although this was the largest study on ADV pneumonia after allo-HSCT, it was retrospective, and the sample size was still relatively small. In addition, for some of the patients, only semiquantitative results were obtained via ADV PCR, which might influence the accuracy of our analysis of the association between ADV PCR level and mortality in patients with ADV pneumonia; these findings should be further confirmed by multicenter, prospective studies in the future. Finally, only 7 patients received BAL because some of the ADV pneumonia patients could not tolerate bronchoscopy. This might impact the accuracy of the analysis of the association between ADV PCR detection in BALF and the outcome of ADV pneumonia, which should be further investigated in further studies.

In summary, we are the first to report the clinical manifestations, prognostic factors, and outcomes of ADV pneumonia after allo-HSCT, and we further confirmed that this was a life-threatening post-HSCT complication. New drugs or cytotherapy methods may help to further improve the clinical outcomes of these patients.

### Electronic supplementary material

Below is the link to the electronic supplementary material.

Supplementary Material 1

## Data Availability

No datasets were generated or analysed during the current study.

## Abbreviations

ADV: Adenovirus
Allo-HSCT: Allogeneic hematopoietic stem cell transplantation
ATG: Antithymocyte globulin
BAL: Bronchoalveolar lavage
BALF: Bronchoalveolar lavage fluid
CMV: Cytomegalovirus
CT: Cycle threshold
CT: Computed tomography
EBV: Epstein–Barr virus
GVHD: Graft-versus-host disease
ICU: Intensive care unit
ISI: Immunodeficiency score index
OS: Overall survival
RSV: Respiratory syncytial virus
RT-PCR: Real-time quantitative polymerase chain reaction
