# Supplementary material for: Clinical manifestations, prognostic factors, and outcomes of adenovirus pneumonia after allogeneic hematopoietic stem cell transplantation
Source: Virol J. 2024 May 14;21:110. doi: 10.1186/s12985-024-02383-1 (PMC11094961; doi:10.1186/s12985-024-02383-1)
Supplement: Supplementary file 1 — Supplementary Material 1 [file 12985_2024_2383_MOESM1_ESM.pdf]

Additional file 1 Univariate Analysis of variables related to different outcomes after the onset of ADV pneumonia in allo-HSCT recipients

	HR	95%*CI*	*P*
**ICU admission after the onset of ADV pneumonia**			
Sex			
Male		1	
Female	0.83	0.30-2.36	0.732
Recipient age			
<median		1	
≥median	0.76	0.27-2.10	0.596
Disease status			
CR1		1	
≥CR2	3.04	1.09-8.51	0.034
Transplant graft			
PB alone		1	
Others	2.20	0.76-6.35	0.146
Number of HLA-A, B, DR mismatches			
1 or 2		1	
3	1.09	0.14-8.31	0.934
Donor-recipient sex-matched grafts			
Male-male		1	
Male-female	1.38	0.37-5.15	0.635
Female-male	1.09	0.27-4.42	0.900
Female-female	0.37	0.04-3.37	0.380
Other (only cord blood infused)	3.59	0.38-33.70	0.264
Donor/recipient relation			
Others		1	

Maternal donor	0.22	0.03-1.67	0.143
Blood group disparity			
Others		1	
Match	0.76	0.27-2.10	0.594
ISI score			
≤ 2 score (low risk)		1	
>2 score (moderate and high risk)	0.64	0.14-2.86	0.559
LOSP score			
0-2 risk factors		1	
3-4 risk factors	2.682	0.94-7.64	0.065
Infused $CD34^+$ cells ($\times 10^6$/kg)			
<median		1	
≥median	2.43	0.82-7.18	0.110
Infused MNC ($\times 10^8$/kg)			
<median		1	
≥median	0.26	0.08-0.83	0.023
Laboratory values at ADV pneumonia onset			
Absolute monocyte count (<0.20 vs ≥0.20×10^9/L)	0.73	0.27-2.02	0.545
BUN (<7.2 vs ≥7.2 mmol/L)	2.08	0.74-5.89	0.167
Albumin (<30.5 vs ≥30.5 g/L)	0.67	0.24-1.85	0.441
LDH (<250 vs ≥250 U/L)	2.27	0.64-8.08	0.207
High level ADV DNAemia (<10^6 copies vs ≥10^6 copies)	5.45	1.55-19.16	0.008
High level ADV positivity in BALF (<10^5 copies vs ≥10^5 copies)	2.12	0.13-35.36	0.600
Corticosteroid dose (<2 vs ≥2 mg/kg/d)	1.40	0.44-4.42	0.567

Treatment of intravenous gamma globulin (yes vs. no)	2.12	0.48-9.43	0.322
Treatment of acyclovir (yes vs. no)	0.81	0.29-2.28	0.689
Treatment of cidofovir (yes vs. no)	1.20	0.43-3.37	0.733
Treatment of ganciclovir (yes vs. no)	0.47	0.11-2.12	0.328
**Need invasive mechanical ventilation**			
Sex			
Male		1	
Female	0.58	0.18-1.89	0.580
Recipient age			
<median		1	
≥median	0.77	0.26-2.30	0.636
Disease status			
CR1		1	
≥CR2	2.83	0.95-8.48	0.063
Transplant graft			
PB alone		1	
Others	1.87	0.60-5.86	0.281
Number of HLA-A, B, DR mismatches			
1 or 2		1	
3	0.94	0.12-7.26	0.953
Donor-recipient sex-matched grafts			
Male-male		1	
Male-female	1.14	0.28-4.56	0.856
Female-male	1.16	0.29-4.69	0.836
Female-female	0	0	0.979
Other (only cord blood infused)	3.19	0.34-29.79	0.308

Donor/recipient relation			
Others		1	
Maternal donor	0.03	0-8.51	0.228
Blood group disparity			
Others		1	
Match	1.03	0.34-3.07	0.965
ISI score			
≤ 2 score (low risk)		1	
> 2 score (moderate and high risk)	0.56	0.12-2.56	0.457
LOSP score			
0-2 risk factors		1	
3-4 risk factors	3.25	1.08-9.81	0.036
Infused $CD34^+$ cells ($\times 10^6$/kg)			
<median		1	
≥median	2.46	0.75-8.04	0.136
Infused MNC ($\times 10^8$/kg)			
<median		1	
≥median	0.32	0.10-1.09	0.068
Laboratory values at ADV pneumonia onset			
Absolute monocyte count (<0.20 vs ≥0.20×10^9/L)	0.70	0.24-2.10	0.528
BUN (<7.2 vs ≥7.2 mmol/L)	2.61	0.87-7.81	0.088
Albumin (<30.5 vs ≥30.5 g/L)	0.68	0.23-2.03	0.490
LDH (<250 vs ≥250 U/L)	3.09	0.68-14.02	0.144
High level ADV DNAemia (<10^6 copies vs ≥10^6 copies)	5.45	1.55-19.16	0.008
High level ADV positivity in BALF (<10^5 copies vs	2.12	0.13-35.36	0.600

≥10^5 copies)			
Corticosteroid dose (<2 vs ≥2 mg/kg/d)	1.15	0.32-4.22	0.830
Treatment of intravenous gamma globulin (yes vs. no)	3.94	0.51-30.37	0.188
Treatment of acyclovir (yes vs. no)	0.76	0.25-2.32	0.626
Treatment of cidofovir (yes vs. no)	1.11	0.36-3.41	0.851
Treatment of ganciclovir (yes vs. no)	0.28	0.04-2.17	0.223
**ADV pneumonia-related mortality**			
Sex			
Male		1	
Female	0.29	0.06-1.35	0.115
Recipient age			
<median		1	
≥median	0.73	0.22-2.41	0.608
Disease status			
CR1		1	
≥CR2	2.17	0.65-7.20	0.207
Transplant graft			
PB alone		1	
Others	4.01	1.18-13.62	0.026
Number of HLA-A, B, DR mismatches			
1 or 2		1	
3	0.79	0.10-6.20	0.823
Donor-recipient sex-matched grafts			
Male-male		1	
Male-female	0.29	0.03-2.63	0.274
Female-male	1.11	0.27-4.50	0.885

Female-female	0.39	0.04-3.58	0.408
Other (only cord blood infused)	3.60	0.38-34.50	0.267
Donor/recipient relation			
Others		1	
Maternal donor	0.31	0.04-2.45	0.266
Blood group disparity			
Others		1	
Match	0.49	0.14-1.69	0.257
ISI score			
≤ 2 score (low risk)		1	
> 2 score (moderate and high risk)	0.44	0.10-2.08	0.303
LOSP score			
0-2 risk factors		1	
3-4 risk factors	4.59	1.38-15.254	0.013
Infused $CD34^+$ cells ($\times 10^6$/kg)			
<median		1	
≥median	3.20	0.84-12.24	0.090
Infused MNC ($\times 10^8$/kg)			
<median		1	
≥median	0.41	0.12-1.42	0.161
Laboratory values at ADV pneumonia onset			
Absolute monocyte count (<0.20 vs ≥0.20$\times 10^9$/L)	0.32	0.09-1.22	0.095
BUN (<7.2 vs ≥7.2 mmol/L)	1.75	0.51-6.01	0.375
Albumin (<30.5 vs ≥30.5 g/L)	0.65	0.20-2.13	0.477
LDH (<250 vs ≥250 U/L)	5.71	0.73-44.90	0.098
High level ADV DNAemia (<10^6 copies vs ≥10^6	6.39	1.43-28.67	0.015

copies)			
High level ADV positivity in BALF (<10^5 copies vs ≥10^5 copies)	2.12	0.13-35.36	0.600
Corticosteroid dose (<2 vs ≥2 mg/kg/d)	2.21	0.64-7.64	0.208
Treatment of intravenous gamma globulin (yes vs. no)	3.23	0.41-25.31	0.263
Treatment of acyclovir (yes vs. no)	0.69	0.20-2.35	0.549
Treatment of cidofovir (yes vs. no)	2.15	0.65-7.04	0.208
Treatment of ganciclovir (yes vs. no)	0.70	0.15-3.26	0.645
**100-day survival after the onset of ADV pneumonia**			
Sex			
Male		1	
Female	1.00	0.37-2.71	0.995
Recipient age			
<median		1	
≥median	0.68	0.25-1.83	0.442
Disease status			
CR1		1	
≥CR2	2.17	0.79-5.92	0.131
Transplant graft			
PB alone		1	
Others	2.08	0.73-5.90	0.169
Number of HLA-A, B, DR mismatches			
1 or 2		1	
3	1.19	0.16-9.06	0.864
Donor-recipient sex-matched grafts			
Male-male		1	

Male-female	1.43	0.38-5.34	0.596
Female-male	1.06	0.26-4.29	0.933
Female-female	0.75	0.14-4.14	0.739
Other (only cord blood infused)	3.83	0.41-36.13	0.241
Donor/recipient relation			
Others		1	
Maternal donor	0.42	0.09-1.85	0.250
Blood group disparity			
Others		1	
Match	0.84	0.31-2.25	0.723
ISI score			
≤ 2 score (low risk)		1	
> 2 score (moderate and high risk)	0.69	0.16-3.08	0.630
LOSP score			
0-2 risk factors		1	
3-4 risk factors	2.41	0.87-6.72	0.092
Infused $CD34^+$ cells ($\times 10^6$/kg)			
<median		1	
≥median	1.60	0.59-4.37	0.356
Infused MNC ($\times 10^8$/kg)			
<median		1	
≥median	0.32	0.11-0.93	0.036
Laboratory values at ADV pneumonia onset			
Absolute monocyte count (<0.20 vs ≥0.20×10^9/L)	0.66	0.24-1.76	0.401
BUN (<7.2 vs ≥7.2 mmol/L)	2.40	0.89-6.48	0.085
Albumin (<30.5 vs ≥30.5 g/L)	0.98	0.36-2.64	0.970

LDH (<250 vs ≥250 U/L)	4.05	0.92-17.89	0.065
High level ADV DNAemia (<10^6 copies vs ≥10^6 copies)	5.45	1.55-19.16	0.008
High level ADV positivity in BALF (<10^5 copies vs ≥10^5 copies)	2.12	0.13-35.36	0.600
Corticosteroid dose (<2 vs ≥2 mg/kg/d)	1.76	0.61-5.09	0.297
Treatment of intravenous gamma globulin (yes vs. no)	4.87	0.64-36.89	0.126
Treatment of acyclovir (yes vs. no)	0.56	0.19-1.60	0.278
Treatment of cidofovir (yes vs. no)	1.39	0.52-3.73	0.518
Treatment of ganciclovir (yes vs. no)	0.68	0.19-2.41	0.549

Abbreviations: ADV=adenovirus; PB=peripheral blood; HLA= human leukocyte antigen; ISI= immunodeficiency scoring index; MNC= mononuclear cells; BUN=blood urea nitrogen; LDH=lactate dehydrogenase; LOSP=late-onset severe pneumonia; ICU=intensive care unit; BALF=bronchoalveolar lavage fluid; HR=hazard ratio; CI=confidence interval.

* All variables were first included in the univariate analysis; only variables with $P < 0.1$ were included in the Cox proportional hazards model with time-dependent variables.
